# Effect of Temperature on the Physical, Electro-Chemical and Adsorption Properties of Carbon Micro-Spheres Using Hydrothermal Carbonization Process

**DOI:** 10.3390/nano8080597

**Published:** 2018-08-06

**Authors:** Zaira Zaman Chowdhury, Bagavathi Krishnan, Suresh Sagadevan, Rahman Faizur Rafique, Nor Aliya Binti Hamizi, Yasmin Abdul Wahab, Ali Akbar Khan, Rafie Bin Johan, Y. Al-douri, Salim Newaz Kazi, Syed Tawab Shah

**Affiliations:** 1 Nanotechnology and Catalysis Research Center (NANOCAT), University of Malaya, Kuala Lumpur 50603, Malaysia; aliyahamizi@um.edu.my (N.A.B.H.); yasminaw@um.edu.my (Y.A.W.); aakhan.um.plasma@gmail.com (A.A.K.); mrafiej@um.edu.my (R.B.J.); yarub@um.edu.my (Y.A.-d.); tawab_shah2003@yahoo.com (S.T.S.); 2 Department of Chemical Engineering, University Malaysia Pahang, Pekan Pahang 26600, Malaysia; beggyk@gmail.com; 3 Centre for Nanotechnology, AMET University, Chennai 603112, India; 4 Centre for Foundation Studies, Department of Physics, National Defense University Malaysia, Kem Sg. Besi, 57000 Kuala Lumpur, Malaysia; 5 Rutgers Cooperative Extension Water Resources Program, Rutgers, The State University of New Jersey; New Brunswick, NJ 08901, USA; rahman.rafique@rutgers.edu; 6 Physics Department, Faculty of Science, University of Sidi-Bel-Abbes, Sidi-Bel-Abbes 22000, Algeria; 7 Department of Mechatronics Engineering, Faculty of Engineering and Natural Sciences, Bahcesehir University, Besiktas, Istanbul 34349, Turkey; 8 Department of Mechanical Engineering, University of Malaya, Kuala Lumpur 50603, Malaysia; salimnewaz@um.edu.my

**Keywords:** catalyst, lignin, holo-cellulose (HC), isotherms, thermodynamics, hydrothermal carbonization (HTC)

## Abstract

This research deals with the effect of the temperature on the physical, thermal, electrochemical, and adsorption properties of the carbon micro-spheres using hydrothermal carbonization (HTC). Until recently, limited research has been conducted regarding the effects of delignification during the HTC process of biomass residues especially *Dimocarpus longan*. In this regard, lignin was first extracted from the lingo-cellulosic waste of Longan fruit peel (*Dimocarpus longan*). The holocellulose (HC) separated from lignin and raw biomass substrates (Longan fruit exocarp/peel powder, LFP) were carbonized at different temperatures using water as the green catalyst. Hydrothermal carbonization (HTC) was performed for both of the samples (LFP and HC) at 200 °C, 250 °C, and 300 °C for 24 h each. The surface morphological structures, the porosity, and the Brunauer-Emmett-Teller (BET) surface area of the prepared micro-spherical carbon were determined. The BET surface areas obtained for HC-based carbon samples were lower than that of the raw LFP based carbon samples. The carbon obtained was characterized using ultimate and proximate analyses. The surface morphological features and phase transformation of the synthesized micro-spherical carbon was characterized by a field-emission scanning electron microscopy (FE-SEM) and X-ray diffraction (XRD) analysis. The results demonstrated that the extraction of lignin could significantly alter the end properties of the synthesized carbon sample. The carbon spheres derived from LFP showed a higher carbon content than the HC-based carbon. The absence of lignin in the holo-cellulose (HC) made it easy to disintegrate in comparison to the raw, LFP-based carbon samples during the HTC process. The carbonaceous samples (LFP-300 and HC-300) prepared at 300 °C were selected and their adsorption performance for Pb (II) cations was observed using Langmuir, Freundlich, and Temkin linear isotherm models. At 30 °C, the equilibrium data followed the Langmuir isotherm model more than the Freundlich and Temkin model for both the LFP-300 sample and the HC-300 sample. The potential of the synthesized carbon microspheres were further analyzed by thermodynamic characterizations of the adsorption equilibrium system.

## 1. Introduction

Ligno-cellulosic biomass is considered to be a renewable, promising, and eco-friendly alternative resource for the preparation of carbon-based chemicals and fuels [1,2]. The existing global supply of carbon depends mainly on fossil fuels (oil, natural gas, and coal), which are limited in quantity. Due to the rapid depletion of fossil energy, using biomass to produce value-added products has elicited great attention from researchers in modern times [3]. Biomass can be converted to yield char or carbon using different thermal, biological, and physio-chemical processes. Among those techniques, hydrothermal carbonization (HTC) is more convenient since it can be performed at comparatively lower temperatures (150 °C to 350 °C) using acids, bases, metallic salts, or water as a “green catalyst” [4,5,6,7,8]. The overall hydrothermal carbonization (HTC) process is performed under autogenously created pressure inside an autoclave. Lot of complex reactions including condensation, hydrolysis, dehydration, and decarboxylation take place to transform the biomass into a carbon-rich substrate with unique properties [8]. Carbonaceous materials can be produced through thermal treatment of the starting materials such as polysaccharides (cellulose), organic molecules (e.g., furfural), or complex materials (e.g., lingo-cellulosic biomass) with water. The subsequent solid products obtained are enriched with carbon and are known as hydrochars or carbon based on carbon proportions. Depending on the preparation condition, these types of carbon can exhibit attractive physical and electrochemical properties [9,10,11,12,13]. The HTC process is carried out frequently to convert mono-saccharides and oligosaccharides to obtain hydrochars or carbon. Carbon micro-spheres, therefore, can be used as a matrix to fabricate porous carbon after activation [14]. It can even be used to prepare carbon-based composites using a metal or metal oxide catalyst [15,16]. Due to the variation in the cellulose and lignin content of several types of biomass residues, the physiochemical properties of hydro-chars or carbon samples prepared from a certain quantity of ligno-cellulosic substrate can vary greatly across different samples.

Thermochemical processes used for an HTC process can convert the compact heterogeneous network of ligno-cellulosic residues into advantageous forms tailored to consumer requirements. The design of an HTC reactor, feedstock composition, reaction parameters (i.e., holding time, temperature, rate of heating, type, and amount of catalyst and pressure), and the physical characteristics of feedstock (particle size and porosities) have a robust impact on the characteristics of the resulting char or carbon samples [17,18,19]. Hydrothermal carbonization involves the application of aqueous reaction medium, which entails using water as a green catalyst. In that case, the wet biomass can be used, which excludes the pre-drying process before the HTC treatment. The HTC process is more flexible than the dry pyrolysis process. The gaseous products formed during the HTC process are mostly dissolved in the slurry containing char-water mixture. 

Cellulose is a complex, crystalline biopolymer made from D-glucose monomers and is the major constituent of raw biomass. Consequently, it is evident that HTC should exemplify an effective technique to transform biomass residues into carbonaceous materials. However, using lingo-cellulosic biomass as a starting precursor is more complex than using simple monosaccharaide or oligosaccharide molecules. The higher degree of structural complexity in biomass can affect the HTC mechanism to a greater extent as well. Biomass contains cellulose, hemicellulose, and a lignin polymer. In plants, the skeleton of the cell walls is built using cellulose. However, the presence of lignin with some substances like pectins, proteins, and extraneous substances provides strength to the plants [20]. Hemicellulose is a type of branched polymer that is amorphous and contains many other saccharides including mannose, xylose, arabinose, and galactose. Lignin is a non-polysaccharide and it is a highly stable poly-phenolic aromatic biopolymer. The phenyl-propane units are cross-linked to give lignin. The cellulosic fractions in biomass are intricately bonded with lignin, which provides strength to the plants. Cellulose and hemicellulose are easy to transform while lignin is difficult to treat because, at low temperatures, it is partially degraded [19]. The HTC process was carried out previously for cellulose, eucalyptus sawdust, and barley straw within the temperature range of 220 °C to 250 °C for a residence period of 2 h to 4 h [6,7]. Microcrystalline cellulose and wood sawdust were used earlier to produce nanoparticles, which were later used for electrochemical hydrogen generation [21]. Due to the coverage of lignin, the cellulose inside the crude biomass could be minimally disintegrated. The aqueous medium of the hydrothermal conditions can only partially degrade the cellulose and hemicellulose fraction. Therefore, lignin’s existence has negative impacts on the effective transformation of cellulose and hemicellulose into the char sample during the HTC process. The effective surface area also decreases in size due to the presence of lignin. Therefore, removal of lignin with some minor constituents like pectin and wax can enhance the internal surface area for the effective transformation of biomass residues [22,23].

Until recently, limited studies have been carried out to observe the characteristics of char or carbon products resulting from HC (cellulose and hemicellulose) after delignification of raw biomass. To the author’s knowledge, no research has been conducted on the extraction of HC from *Dimocarpus longan* fruit peel and the synthesis of the spherical carbonaceous materials from the raw peel (LFP) using an HTC technique. *Dimocarpus longan* is commonly known as the longan. It grows abundantly in Asia predominantly in China, Taiwan, Vietnam, and Thailand [24]. In 2010, China produced 1300 million tons of longan. The statistics showed that Vietnam and Thailand also produced approximately 600 million and 500 million tons, respectively [25]. The plants grown in the tropical region belongs to the family of *Sapindaceae*. The peel or exocarp of longan fruit is lightweight and thin. After extraction of the juicy edible pulp that is mesocarp, the peels are thrown off. Usage of this waste lingo-cellulosic residue for the synthesis of carbon can solve the waste disposal problem as well as add some valuable products for further application in versatile fields. In this work, HC was extracted from LFP and used as the starting material to prepare solid carbon or char using the HTC process. The morphological as well as electrochemical properties with energy content of the HTC-based carbon samples derived from LFP and HC were studied at temperatures of 200 °C, 250 °C, and 300 °C. The purpose of this study is to evaluate the impact of eliminating lignin and other minor constituents from the biomass, which ultimately can have significant influence on the properties of the carbon sample obtained by the HTC process using water as a “green catalyst”. The synthesized carbon microspheres from LFP and HC were used for adsorption of Pb(II) cations from the aqueous system. Equilibrium isotherm and thermodynamic characterizations were done to determine the parameters influencing the sorption process at different temperatures. 

## 2. Results and Discussions

### 2.1. Effect of Temperature on Physical, Electrochemical, and Thermal Properties of Hydro-Char

#### 2.1.1. Yield, Energy Content, pH, Electrical Conductivity, and Ultimate Analysis

Table 1 illustrates the CHNOS analysis of the precursors (HC and LFP) along with the synthesized sample at different temperatures. A progressive increase in temperature during the HTC process raised the carbon content of a sample, but decreased the oxygen and hydrogen content [26,27,28]. The H/C and O/C ratios decreased after carbonization at higher temperatures for both of the samples. The decreased H/C ratio was ascribed to the increased rate that the aromatization reactions occurred during the HTC process [29]. Due to a decarboxylation reaction, the O/C ratio was decreased. The H/C and O/C ratios decreased due to dehydration [30]. The H/C and O/C ratios in the HC-based carbon samples decreased more noticeably than those with the LFP powder. This was due to the higher coalification values for the HC-based samples. The yield of the carbon samples was inversely proportional with the carbonization temperature. The yield dropped significantly after the temperature from 200 °C to 300 °C for both the precursors increased (HC and LFP), which is illustrated by Table 1. Thermal degradation of lingo-cellulosic biomass usually occurs at lower temperatures. At higher temperatures, the higher molecular weight organic compounds in the biomass are devolatilized into lower molecular weight compounds along with some gases being produced as well [31]. Dehydration and elimination reactions would proceed faster at higher temperatures, which leads to a decreased char yield for both samples [32,33,34]. This trend was anticipated since the mass-yield loss at higher temperatures was associated with progressive dehydrogenation and aromatization of the hydrochar sample, which included the subsequent decomposition of inorganic elements [19]. The HTC process is considered an efficient way for densification of the energy of the biomass matrix [35]. From Table 1, it can be observed that the energy content (HHV) of the carbon samples increased with rising HTC temperatures. However, the HC-based carbon samples showed slightly higher values of HHV than the LFP samples. The pH values obtained for the LFP samples were slightly more acidic than the HC-based samples. The electrical conductivity (EC) increased slightly with rising temperatures, which indicated a high salinity for all of the samples. The results aligned with the previous work conducted on the hydrothermal carbonization of rice husk [36] where the magnitude of the pH and EC values of the synthesized carbon samples somewhat enhanced with an increasing rate of temperature. This was expected due to the existence of a larger amount of ash residue in the char or carbon sample produced at a higher carbonization temperature [37].

#### 2.1.2. Thermogravimetric/Proximate Analysis

**Figure 1:**
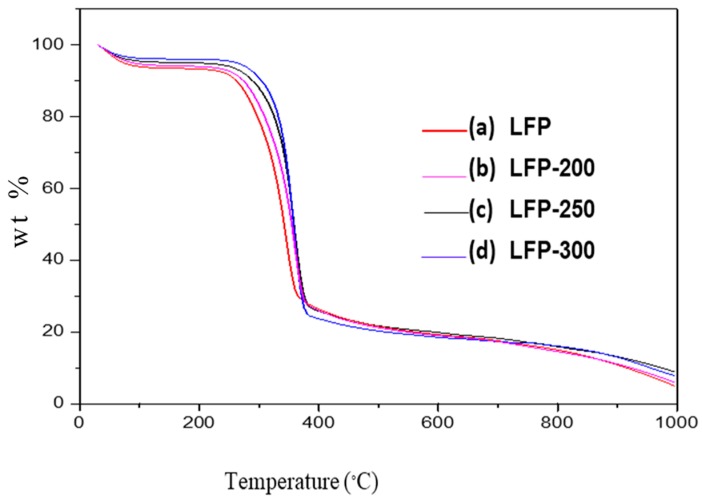
Thermogravimetric curves for (**a**) Raw *Dimocarpus longan* peelpowder (LFP) and their corresponding carbon micro-spheres produced at various temperatures (**b**) LFP-200; (**c**) LFP-250; and (**d**) LFP-300.

**Figure 2:**
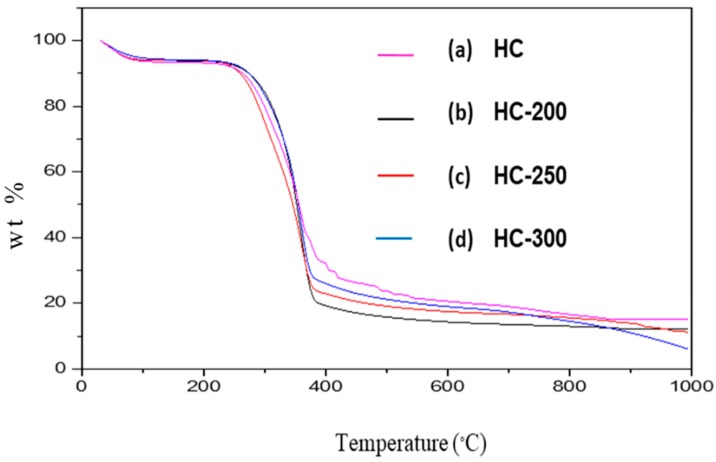
Thermogravimetric curves for (**a**) Raw Holocellulose (HC) and corresponding carbon micro-spheres produced at various temperatures (**b**) HC-200; (**c**) HC-250; and (**d**) HC-300.

The thermal stabilities of both LFP and HC samples with their corresponding carbon spheres were investigated using the thermo-gravimetric method and are shown in Figure 1 and Figure 2 for LFP- and HC-based carbon, respectively.

The TGA curve showed several stages for the synthesized samples. The initial degradation step occurred at approximately 80 °C to 120 °C. This was due to the loss of the adsorbed moisture. The subsequent degradation step for LFP occurred between about 200 °C to 300 °C and represented the degradation of hemicellulose. At temperatures between 300 °C and 400 °C, cellulose degradation occurred. It was previously described that the degradation of hemicelluloses somewhat overlapped with the cellulose degradation in biomass. Due to the complex structure of lignin, the degradation of this macromolecule took place within the long temperature range, which started at 200 °C until 800 °C [38,39]. The presence of the phenyl group made the decomposition of the lignin much more difficult [19]. Compared with untreated LFP, the carbon sample derived from LFP showed higher thermal stability at different temperatures. This phenomenon was earlier observed for the HTC conversion of poplar wood-based samples [30].

As illustrated by Table 2, the DTG_max_ of LFP-200, LFP-250, and LFP-300 were 342.37 °C, 355.77 °C, and 369.89 °C, respectively. The amorphous domain of the cellulosic chain as well as the hemicellulose fraction was removed during the hydrothermal carbonization (HTC) process of the LFP samples, which resulted in a higher thermal stability for the prepared carbon samples. With a subsequent increase in temperature, the sample contained a greater proportion of a heat resistant stable form of carbon particles. This was further confirmed by an elemental analysis of the synthesized samples. The percentage of carbon in the LFP sample was initially low, but, after consecutive treatment using the HTC process, the percentage of carbon increased with a rise in temperature (Table 1). The thermal stability of the HC-based hydrochars grew with increasing temperatures. The carbon content in these samples also rose due to an elevated temperature (Table 1). The ash content of the synthesized samples was higher than the raw lingo-cellulosic biomass sample. Compared to LFP-200, LFP-250, and LFP-300, the HC-based samples had more ash and fixed carbon content. At the same time, the volatile component in these samples decreased with an increased temperature. Analogous tendency was observed for the HTC-based carbon as well.

#### 2.1.3. Surface Morphology Analysis

**Figure 3:**
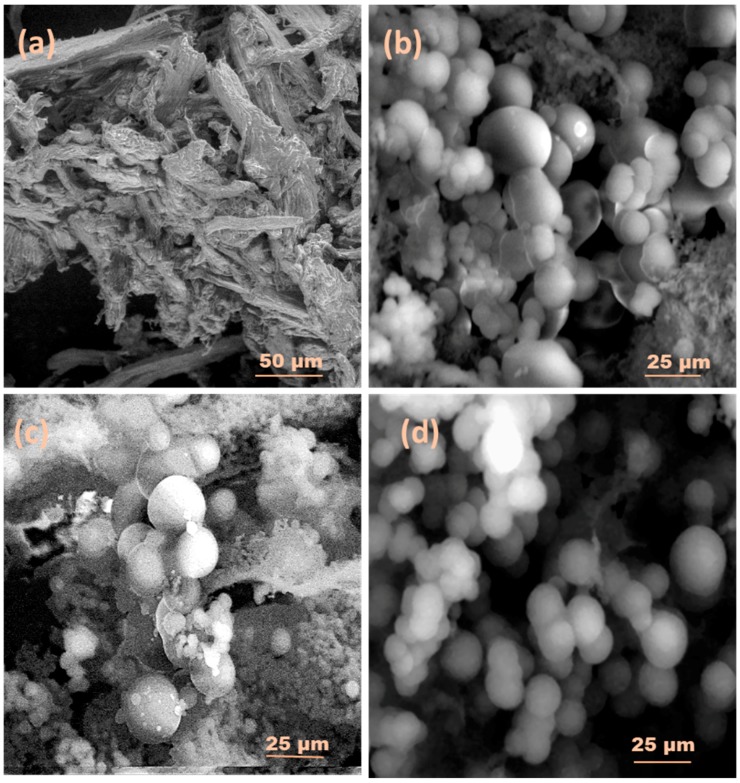
SEM images of (**a**) Raw *Dimocarpus longan* peel powder (LFP) and their corresponding carbon micro-spheres (**b**) LFP-200; (**c**) LFP-250; and (**d**) LFP-300 produced at various temperatures.

**Figure 4:**
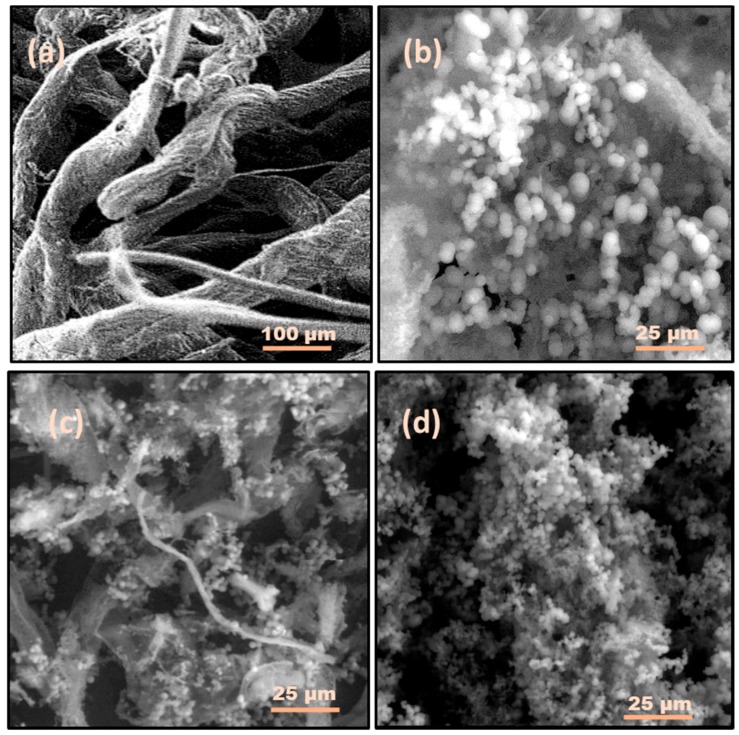
SEM images of (**a**) Raw Holocellulose (HC) and corresponding carbon micro-spheres: (**b**) HC-200; (**c**) HC-250; and (**d**) HC-300 produced at various temperatures.

The SEM images of raw LFP with extracted HC and their respective carbon samples produced at different temperatures are illustrated by Figure 3a–d and Figure 4a–d, respectively. The surface of the wood powder was comparatively rough with some cracks. However, after hydrothermal carbonization (HTC) at different temperatures, some micro-spherical carbon was deposited on the surface of the LFP. The HTC process degraded the cellulose and hemicellulose components substantially to produce carbon microspheres on the surface of the raw peel powder [30,39]. Nevertheless, the lignin content of the biomass was difficult to degrade at lower temperatures [6,9,21]. With an increasing temperature, the number of carbon microspheres rose but the size of the carbon microspheres was reduced. At lower temperatures, the LFP particles were fragmented and showed the development of comparatively large, irregularly-shaped carbon microspheres (Figure 3b; see the LFP-200 sample). Comparatively, more carbon microspheres were formed on the surface of the samples called LFP-250 and LFP-300. 

The HC extracted from the LFP powder was fibrillated. The entire skeleton of the extracted HC was covered by carbon microspheres of varying sizes. The proportion of these carbon microspheres increased successively with a growth in temperature from 200 °C to 300 °C during the HTC process (HC-200, HC-250, and HC-300). Compared to LFP-based carbon samples, the number of carbon microspheres in HC-based samples was greater. Furthermore, the sizes of the carbon microspheres were comparatively smaller for HC-based samples. This clearly reflected the presence of lignin in LFP, which made it more difficult to be carbonized completely in comparison with the HC, which contained only a mixture of cellulose and hemicellulose.

#### 2.1.4. Surface Area and Porosity Analysis

**Figure 5:**
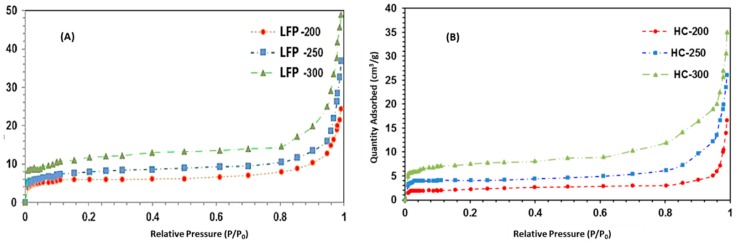
BET Isotherms of (**A**) LFP-200, LFP-250, and LFP-300; (**B**) HC-200, HC-250, and HC-300.

Figure 5A,B display the N_2_ adsorption isotherms for LFP-based and HC-based hydrochars, respectively. The surface area of HC-based carbon samples was slightly less. HC-based carbon spheres had poor porosity in comparison with the LFP-based samples. At lower temperatures, both the samples showed comparatively reduced surface areas, which was due to the inadequate carbonization. However, the isotherm obtained for both types of carbon samples were obtained at higher temperatures, which could be classified as an intermediate between type I and type II isotherms [40]. Near the relative pressure of approximately 0.9, the curve showed an upward trend. This indicated that the obtained carbon samples contained virtually no framework of confined pores [41]. The presence of inter-particle voids caused an increase in the surface area [42]. The type of starting material and temperature of the process can control the amount of pores due to the cracking of the basal structural sheets of the carbon particles [43,44]. Due to incomplete carbonization at lower temperatures during the HTC process, the pores were clogged by the tarry substances. Treatment at higher temperatures caused selective elimination of those components by volatilization, which resulted in a higher pore volume. Higher temperatures further led to de-polymerization reactions, which increased the surface area of both types of carbon spheres [45].

The textural parameters determined for both starting materials with corresponding carbon micro-spheres are listed in Table 3.

#### 2.1.5. Crystallinity or X-ray Diffraction (XRD) Analysis

**Figure 6:**
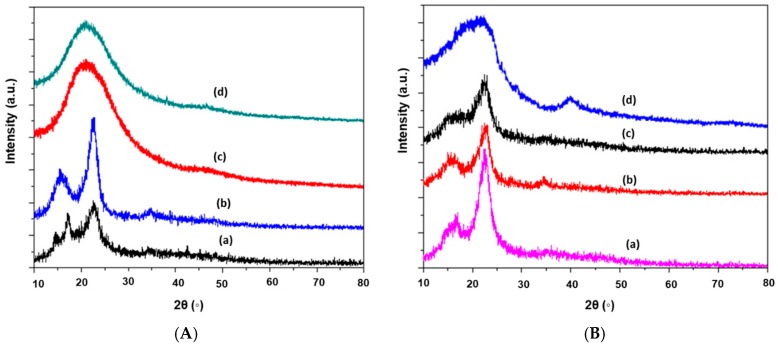
(**A**) X-ray Spectra of *Dimocarpus longan* peel powder (LFP) and the corresponding carbon micro-spheres produced at various temperatures: (**a**) LFP Powder; (**b**) LFP-200; (**c**) LFP-250; and (**d**) LFP-300 produced at various temperatures; (**B**) X-ray Spectra of HC and their corresponding carbon micro-spheres produced at various temperatures: (**a**) HC; (**b**) HC-200; (**c**) HC-250; and (**d**) HC-300.

The XRD spectra for the raw LFP sample with the extracted HC and their corresponding carbon produced at different temperatures are illustrated by Figure 6A,B, respectively. The two sharp narrow peaks in Figure 6A,B at 2θ values of around 16.5° and 22.6° show the presence of amorphous and crystalline regions of cellulose in the raw LFP and HC samples. The crystallinity obtained for HC was 58.68%, which was higher than that of the LFP peel powder (46.28%). The increasing trend of crystallinity in HC was due to the delignification and the partial removal of hemicellulose from the LFP powder during the preparation of HC. When both the LFP and HC samples were hydrothermally carbonized, the resulting carbon showed different XRD patterns. When the temperature increased from 200 °C to 300 °C, the peaks around 22° to 24° broadened in both the samples of LFP-based and HC-based carbons.

For LFP-based and HC-based carbons, the intensities of these two peaks decreased slightly with a rise in temperature [46]. The amorphous texture of the prepared carbon samples (LFP-300, HC-250, and HC-300) was confirmed by the absence of a sharp peak in the XRD spectra [47]. The XRD patterns of LFP-based carbon showed that hydrothermal carbonization at lower temperatures could not disrupt the crystalline texture of cellulose completely. In prior research, this phenomenon was similarly observed for sawdust-based, wheat straw-based, and corn stalk-based hydro-carbon and potassium hydroxide (KOH)-treated hydro-char [42]. 

Compared to LFP-250, the sharp peak around 16° was completely absent in HC-250. The presence of lignin in LFP-250 made it difficult for the sample to be carbonized more completely than the HC-250 sample. 

### 2.2. Equilibrium Adsorption Isotherm and Thermodynamic Parameters Evaluation

The adsorbent-adsorbate reaction mechanism under the equilibrium system can be illustrated by using different isotherm models. In this research, a linear regression analysis was carried out for synthesized carbon microspheres at different temperature for both types of hydrochar samples. The linear form of the Langmuir isotherm can be represented by the equation below [47].

(1) $$\frac{C_{e}}{q_{e}}=\frac{1}{q_{\max}K_{L}}+\frac{1}{q_{\max}}C_{e}$$

In Equation (1), *q_m_* (mg/g) represents the maximum monolayer adsorption capacity. *C_e_* (mg/L) is the equilibrium concentration of the adsorbate at a liquid phase and *q_e_* (mg/g) is the equilibrium amount of adsorbate adsorbed over the adsorbent’s surface. *K_L_* is the Langmuir adsorption constant (L/mg) related to the binding energy for sorption. *R_L_* is the separation factor obtained from the Langmuir equation [47].

(2) $$R_{L}=\frac{1}{1+K_{L}C_{0}}$$

For this study, *R_L_* values are determined for a maximum initial concentration under investigation (150 mg/L). If the magnitude of RL values are within 0 to 1, then the adsorption process is favorable.

The Freundlich isotherm is frequently used to illustrate the surface heterogeneity of the process with multilayer formation over the surface [48]. The linear form of the Freundlich isotherm is shown below.

(3) $$\ln q_{e}=\ln K_{f}+\frac{1}{n}\ln C_{e}$$

In Equation (3), *K_f_* (mg/g) is the affinity factor of the adsorbate towards the adsorbent and 1/*n* represents the intensity of adsorption, respectively [48].

Table 4 summarizes the values obtained for isotherm parameters in LFP-300 and HC 300 samples.

The linear form of the Temkin Isotherm can be expressed by the equation below [45].

(4) $$q_{e}=\frac{RT}{b}\ln K_{T}+\frac{RT}{b}\ln C_{e}$$

In this paper, *RT*/*b* = *B* (J/mol), denotes the Temkin constant, which depicts the heat of the sorption process while *K_T_* (L/g) reflects the equilibrium binding constant. *R* (8.314 J/mol k) is the universal gas constant and T° (K) is the absolute solution temperature [49].

**Figure 7:**
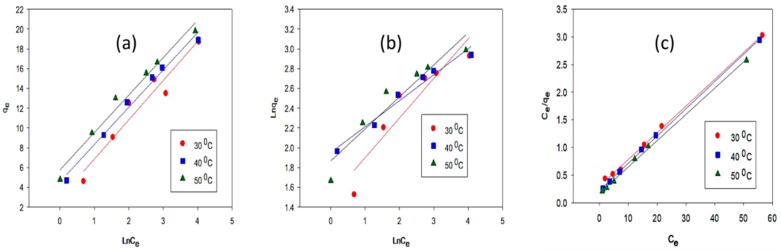
Linear Regression Analysis of (**a**) Langmuir; (**b**) Freundlich; and (**c**) Temkin Isotherm model at different temperature for LFP-300.

**Figure 8:**
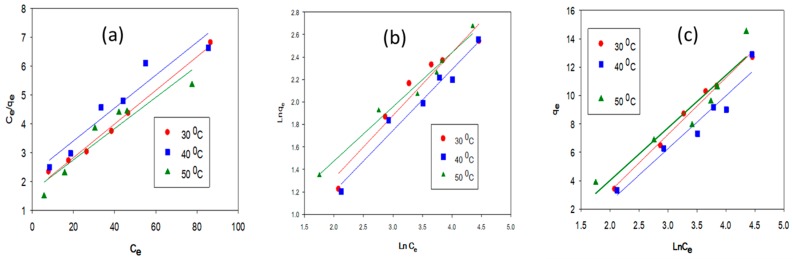
Linear Regression Analysis of (**a**) Langmuir; (**b**) Freundlich; and (**c**) Temkin Isotherm model at different temperatures for LFP-300.

The magnitude of the Langmuir separation factor, *R_L_*, and the Freundlich exponent 1/*n* are below one (Table 4). This represents favorable adsorption processes [50]. Furthermore, the Langmuir monolayer adsorption capacity (*q*_max_) grew with a successive increase of the temperature from 30 °C to 50 °C, which showed the endothermic nature of the sorption process [50,51]. Similar trend have been followed by untreated and acid-activated corncob biomass for the sorption of divalent Mn^2+^ cations [51]. It was further validated by thermodynamic characterization of the system. The linear regression obtained for different isotherm models is illustrated by Figure 7 and Figure 8.

Thermodynamic parameters including Gibbs free energy (∆*G*°), enthalpy (∆*H*°), and entropy (∆*S*°) of the equilibrium system were evaluated for LFP-300 and HC 300 [52]. The linear equations used for this are shown below.

(5) $$Ln\;K_{L}=\frac{\Delta S}{\Delta R}-\frac{\Delta H}{RT}$$

(6) $$\Delta G=RT\;Ln\;K_{L}$$

In the equations above, constant *K_L_* (L/mg) was earlier determined using the Langmuir equation at three different temperatures. *R* is the universal gas constant (8.314 J/ mol·K) and *T* is the absolute temperature in Kelvin scale. The ln*K_L_* versus the 1/*T* plot was used to calculate thermodynamic parameters and it is listed in Table 5. The endothermic property of the equilibrium system was observed as the magnitude of the enthalpy (∆*H*°) obtained, which is positive [50,53,54]. As such, with increasing temperatures, the adsorption percentages will grow. The entropy, ∆*S*°, determined was also positive. This reflected the increased degree of freedom as well as the enhanced randomness at the adsorbent-adsorbate interface. Gibbs-free energy change, ∆*G*°, was negative. Therefore, it can be concluded that the sorption process observed in this scenario for LFP-300 and HC-300 was feasible and spontaneous [52]. 

## 3. Materials and Methods

### 3.1. Grinding and Sieving of Dimocarpus longan Peel (LFP)

The fruits of *Dimocarpus longan* were purchased from a local market in Malacca, Malaysia. The mesocarp of the fruits were separated from the peel or exocarp of the longan fruits and then ground to powder. To eliminate the larger particles, the grounded LFP samples were passed through a 20 µm mesh screen. After screening, the LFP powder was washed thoroughly with deionized water and dried at 90 °C for 24 h. The raw samples were packed in closed bottles for the initial characterization. 

### 3.2. Delignification of Dimocarpus longan Peel (LFP)

The pectin, gum, and wax were first removed from the LFP powder by using a Soxhlet extractor (Z556203, Sigma Aldrich, and Kuala Lumpur, Malaysia). At first, the LFP powder was mixed with a 2:1 solution of benzene/ethanol (*v*/*v*) solvent at 75 °C for 4 h. The sample was filtered and dried for 6 h at 60 °C. To ensure complete delignification, microwave assisted alkali pretreatment was carried out. 2.5 M NaOH was mixed with the sample where the LFP powder to solvent ratio was kept at 1:30 under microwave irradiation. The power of the microwave was kept constant at 150 W. Under microwave irradiation, it was kept for 90 min at 65 °C. After cooling, the filter cake was washed with de-ionized water to remove excess alkali and dried in an oven at 55 °C for 4 h. The light brown mass (1 gm) obtained was bleached using 50 mL solution of 30% hydrogen peroxide (H_2_O_2_) for 4 h at 60 °C. Then the sample obtained was labeled as holocellulose (HC) and was repetitively washed again with hot deionized water and oven-dried at 80 °C. The HC sample obtained was stored in an airtight container for further characterization.

### 3.3. Hydrothermal Carbonization of HC and LFP

A total of 5 g of holocellulose (HC) and raw LFP powder was added to 50 mL of deionized water. The mixture was subsequently placed inside a Teflon-lined steel autoclave with a 100-mL capacity. The mixture was sealed and heated at temperatures ranging from 200 °C to 300 °C for 24 h. The mixture was allowed to cool at room temperature. The resulting carbonaceous materials were filtered off and washed several times with deionized water. Lastly, the sample was oven-dried at 80 °C for 6 h. The resulting sample was labeled according to the starting precursors and treatment temperatures used, which included LFP-200, LFP-250, and LFP-300 for the raw *Dimocarpus longan* peel samples and HC-200, HC-250, and HC-300 for HC-based carbon samples, respectively.

### 3.4. Synthesis of Carbon Micro-Spheres and Physical and Electro-Chemical Characterizations

The raw biomass of *Dimocarpus longan* (LFP) peel and the extracted HC along with the prepared hydrochar samples were characterized by a CHNOS elemental analyzer (PerkinElmer-2400, Tokyo, Japan). Next, thermo-gravimetric analysis (TGA) coupled with a differential thermal analyzer (DTA; Mettler Toledo Star SW901, Tokyo, Japan) was performed to check the thermal stability of the samples under a nitrogen flow rate of 5 mL/min. For the TGA analysis, 5 mg of each sample were taken and heated to 800 °C at a heating rate of 5 °C/min. The fixed carbon content after TGA analysis was estimated by using Equation (7) [55].

Fixed Carbon (%) = 100 − (Moisture % + ash % + Volatile matter %)(7)

The solid carbonaceous product yield after hydrothermal carbonization was determined using Equation (8).

(8) $$\mathrm{Yield}\;\left(\%\right)=\frac{W_{2}}{W_{1}}\times 100$$

where, W_1_ is the dry weight of the starting sample prior to the treatment (g) and W_2_ is the final carbonaceous substances’ weight (g) [36].

The theoretical higher heating value (*HHV predicted*) was calculated using an equation from Channiwala and Parikh (2002). Equation (9) is shown below.

(9) $$HHV_{predicted}(MJKg^{-1})=0.3491C+1.1783H+0.1005S-0.1034O-0.0015N-0.0211A$$

where, C, H, S, O, N, and A represent the weight percentages of carbon, hydrogen, sulfur, oxygen, nitrogen, and ash in carbon, respectively [56]. This equation has been used by previous researchers and the relative error between the calculated and predicted values is less than 6% [57,58]. The pH and electrical conductivity (EC) were measured using the method described in other literature [59]. At first suspensions of 0.01 M calcium chloride, CaCl_2_ and distilled H_2_O (1:5 *v*/*v*) were prepared and 1 g of carbon sample was added. The mixtures were shaken for 1 h on a low-speed shaker at room temperature. After sedimentation of the carbonaceous material for an additional hour, the pH and EC were determined in the supernatant [32]. X-ray diffraction (XRD) patterns of the raw samples and the carbon samples were examined using a D5005 (Bruker, Berlin, Germany) at 40 kV and 40 mA with Cu and Kα radiation. The percentage crystallinity can be calculated by using the equation below [60,61].

(10) $$C=\frac{I_{002}-I_{am}}{I_{002}}\times 100$$

where, *I*_002_ is the maximum intensity of peak 002 at 2θ = 22.5°, *I_am_* is the intensity at 2θ =18.0°, and *C* is the crystallinity index [60,61]. 

A scanning electron microscope (SEM, Model Leo Supra50VP Field Emission, London, UK) was used to obtain the morphological features of the prepared carbons with the raw wood sample. The BET surface area, pore volume, and pore size distribution of the prepared carbon spheres were analyzed with an Autosorb1: Quantachrome Autosorb automated gas sorption system supplied by Quantachrome (Berlin, Germany). The BET equation was used to calculate the surface area of the hydrochar. 

### 3.5. Equilibrium Adsorption Isotherm and Thermodynamics Studies

50 mL solution of Pb(II) cations with concentrations of 25 ppm, 50 ppm, 70 ppm, 90 ppm, 100 ppm, and 150 ppm were placed inside the conical flasks. A total of 0.25 g of synthesized carbon microspheres were added to the solution. The solutions were agitated at 200 rpm for different temperatures of 30 °C, 40 °C, and 50 °C. The pH of the water samples were adjusted to 5.5. After the system has reached equilibrium within 6 h, the water samples were withdrawn and filtered to separate the carbon sample. The filtrate was analyzed to measure residual metal ion concentration. The following equation was used to calculate the amount adsorbed onto the surface of the carbon spheres [50,52,53,54].

(11) $$q_{e}=\frac{(C_{0}-C_{e})V}{W}$$

In the equation above, *q_e_* (mg/g) shows the amount of ion adsorbed onto the surface of the carbon at equilibrium. *C*_0_ is the initial metal ion concentration and *C_e_* (mg/L) is the liquid-phase concentrations of Pb(II) ions at equilibrium conditions. *V* (L) is the volume of the synthetic solution and *W* (g) is the mass of the carbon sample [50,52]. Experimental data was fitted with the linear isotherm models of Langmuir, Freundlich, and Temkin. Thermodynamic calculation was done to determine the values of ∆*G*, ∆*H*, and ∆*S* of the equilibrium system using the Sigma Plot, version 15.

## 4. Conclusions

This study investigated the potential of raw *Dimocarpus longan* peel (LFP) and HC made from the LFP to produce micro-spherical carbon using a hydrothermal carbonization technique. The effect of the carbonization temperature on the energy content as well as physical and electro-chemical properties of the synthesized carbon was evaluated. The carbon yield decreased greatly for both the precursors when the HTC temperature was increased from 200 °C to 300 °C. The HTC temperature extensively influenced the physicochemical characteristics of the prepared carbon spheres. An increase in temperature enhanced the proportion of the carbon substantially while the oxygen and hydrogen content of the carbon sample decreased. A rise in temperature enhanced the BET surface area and changed the porous texture of the synthesized carbon sample. This showed that porosity was not developed sufficiently during the HTC process. The XRD pattern clearly revealed that the crystalline region of the cellulose in the HC-based carbon spheres was destroyed at 250 °C. The crystalline structure of the cellulose in the HC-based char can be easily destroyed and carbonized without the shielding effect of lignin. The amorphous texture of carbon was observed at 300 °C for the LFP-based carbon sample. After delignification, the HC extract showed less thermal stability than the LFP peel powder. The presence of the hemicellulose and cellulose biopolymer embedded inside the lignin was more challenging to degrade thermally. The high aromaticity of lignin provided resistance for the LFP powder, which was easily degraded. The amount of energy content determined in HC-derived carbon spheres was greater than the LFP peel powder based on HTC samples. Equilibrium data were well fitted with the Langmuir isotherm for LFP-300 and HC-300. The Langmuir monolayer adsorption capacity rose with the increasing trend of temperature. LFP-300 had shown more monolayer adsorption capacity than the HC-300 sample due to its larger surface area. The thermodynamic profile of the equilibrium system suggested an endothermic nature as well as feasibility of the sorption process.

## Figures and Tables

**Table 1:** Properties of *Dimocarpus longan* fruit peel powder (LFP) and Holocellulose (HC) with their carbonized samples at different temperatures.

Sample	Ultimate or CHNOS Analysis (wt %)							Yield	HHV	pH	EC
	C	H	N	O	S	H/C	O/C	%	MJ-Kg^−1^	-	mS
Raw LFP	32.65	10.87	2.12	54.34	0.02	0.33	1.66	-	18.05	-	-
LFP-200	40.78	7.66	2.02	49.51	0.03	0.19	1.21	56.45	18.95	4.92	0.97
LFP-250	52.21	4.67	1.23	41.87	0.02	0.09	0.80	43.89	19.09	5.79	1.48
LFP-300	70.88	3.65	1.01	26.45	0.01	0.05	0.37	33.32	25.56	5.99	1.72
Raw HC	32.77	10.40	6.06	50.74	0.04	0.32	1.55	-	18.46	-	-
HC-200	41.88	8.35	5.12	44.63	0.02	0.20	1.06	57.45	19.61	4.85	0.91
HC-250	53.99	5.01	2.01	38.96	0.03	0.09	0.72	48.98	20.44	5.65	1.23
HC-300	71.99	3.02	0.55	24.41	0.03	0.04	0.34	35.89	25.86	5.98	1.55

**Table 2:** Proximate Analysis of LFP Powder and HC with their Carbonized Samples Resulting from HTC at Different Temperatures.

Sample	Proximate Analysis (wt %)				*DTG*_Max_
	Water Content	Volatile Matter	Fixed Carbon	Ash	
Raw LFP	8.07	77.88	3.38	10.67	334.78
LFP-200	6.78	40.67	41.54	11.01	342.37
LFP-250	4.25	30.32	52.23	13.20	355.77
LFP-300	2.89	10.78	71.55	14.78	369.89
Raw HC	9.23	76.45	2.99	11.33	324.89
HC-200	6.66	39.23	40.12	13.99	335.98
HC-250	5.89	23.32	55.87	14.92	349.90
HC-300	2.12	11.88	69.33	16.67	357.99

**Table 3:** Pore characteristics of *Dimocarpus longan* peel powder (LFP) and Holocellulose (HC) with carbonized samples resulting from hydrothermal treatment (HTC) at different temperatures.

Sample	BET Surface Area (m^2^/g)	BJH Cumulative Adsorption Surface Area (m^2^/g)	Total Pore Volume × 10^−3^ (cm^3^/g)	Average Pore Diameter (nm)
Raw LFP	3.02	1.04	0.022	0.56
LFP-200	9.76	8.88	0.036	3.23
LFP-250	13.65	11.09	0.055	4.98
LFP-300	18.32	15.77	0.068	8.98
Raw HC	1.51	1.02	0.013	0.22
HC-200	5.98	4.77	0.021	1.23
HC-250	7.12	5.43	0.034	2.12
HC-300	10.76	8.88	0.046	5.67

**Table 4:** Isotherm Model parameters at Different Temperature for LFP-300 and HC-300.

Sample	Temperature	Langmuir Isotherm				Freundlich Isotherm			Temkin Isotherm		
	°C	*q*_max_ (mg/g)	*K_L_* (L/mg)	*R_L_*	*R* ^2^	*K_F_* (mg/g) (L/mg)^1/*n*^	1/*n*	*R* ^2^	*B*	*K_T_* (L/mg)	*R* ^2^
LFP-300	30	20.40	0.1658	0.038	0.998	4.474	0.401	0.879	3.964	4.5815	0.935
	40	21.27	0.2143	0.030	0.999	7.027	0.263	0.967	3.766	25.621	0.986
	50	22.72	0.3135	0.021	0.998	6.461	0.324	0.907	3.781	84.576	0.978
HC-300	30	17.03	0.1691	0.035	0.992	1.229	0.534	0.932	4.002	3.2861	0.989
	40	17.54	0.2132	0.026	0.918	1.122	0.584	0.968	3.727	3.7488	0.933
	50	18.59	0.3015	0.032	0.909	1.052	0.434	0.978	3.702	2.491	0.903

**Table 5:** Thermodynamic parameters of Pb (II) adsorption for LFP-300 and HC-300.

Sample	Temperature, °K	∆*G*° (KJ-mol^−1^)	∆*H*° (KJ-mol^−1^)	∆*S*° (JK^−1^ mol^−1^)	*R* ^2^
	303	−4.526	+25.870	+0.0702	0.9821
LFP-300	323	−4.009			
	343	−3.118			
	303	−4.477	+23.462	+0.0624	0.981
HC-300	323	−4.021			
	343	−3.219			
